# Association of Red Blood Cell Distribution Width Levels with Connective Tissue Disease-Associated Interstitial Lung Disease (CTD-ILD)

**DOI:** 10.1155/2021/5536360

**Published:** 2021-08-06

**Authors:** Shenyun Shi, Ling Chen, Xianhua Gui, Lulu Chen, Xiaohua Qiu, Min Yu, Yonglong Xiao

**Affiliations:** ^1^Department of Respiratory and Critical Care Medicine, Nanjing Drum Tower Hospital, The Affiliated Hospital of Nanjing University Medical School, No. 321 Zhongshan Road, Nanjing, 210008 Jiangsu, China; ^2^Department of Respiratory and Critical Care Medicine, Nanjing Drum Tower Hospital, Clinical College of Nanjing Medical University, Nanjing, 210008 Jiangsu, China

## Abstract

**Objective:**

The aim of this study was to evaluate the diagnostic and prognostic value of red blood cell distribution width (RDW) in patients with connective tissue disease-associated interstitial lung disease (CTD-ILD).

**Methods:**

We retrospectively reviewed 213 CTD-ILD patients and 97 CTD patients without ILD from February 2017 to February 2020. Hospital and office records were used as data sources. CTD-ILD patients were followed up.

**Results:**

Patients with CTD-ILD had significantly higher RDW than those with CTD without ILD (*p* < 0.001). The area under the receiver operating characteristic curve (AUROC) of RDW for discriminating CTD-ILD from CTD without ILD was 0.64 (95% CI: 0.57-0.70, *p* < 0.001). The cutoff value of RDW for discriminating CTD-ILD from CTD without ILD was 13.95% with their corresponding specificity (55.9%) and sensitivity (70.1%). Correlation analyses showed that the increased RDW was significantly correlated with decreased DLCO%predicted (*r* = −0.211, *p* = 0.002). Cox multiple regression analysis indicated that RDW (HR = 1.495, *p* < 0.001) was an independent factor in the survival of CTD-ILD. The best cutoff value of RDW to predict the survival of patients with CTD-ILD was 14.05% (AUC = 0.78, 95% CI: 0.72-0.84, *p* < 0.001). The log-rank test showed a significant difference in survival between the two groups (RDW > 14.05% and RDW < 14.05%).

**Conclusion:**

RDW was higher in CTD-ILD patients and had a negative correlation with DLCO%predicted. RDW may be an important serum biomarker for severity and prognosis of patients with CTD-ILD.

## 1. Background

Interstitial lung diseases (ILDs) are a group of diseases in which characterized by varying degrees of inflammation and fibrosis of lung parenchyma and interstitium. ILD is strongly associated with various connective tissue diseases (CTDs), such as rheumatoid arthritis (RA), systemic lupus erythematosus (SLE), systemic sclerosis (SSc), systematic vasculitis, polymyositis/dermatomyositis (PM/DM), Sjögren's syndrome (SS), mixed CTD (MCTD), and undifferentiated CTD (UCTD) [1, 2]. The presence of ILD is common in CTD, which is associated with reduced quality of life and a leading cause of mortality [3]. Although it has been reported that five-year survival was 80% and median survival was approximately 12.6 years in a cohort of CTD-ILD [4], progression and prognosis of CTD-ILD vary widely between CTD subtypes. In cases of rapidly progressing and severe CTD-ILD, sensitive recognition and appropriate treatment of CTD-ILD could be critical to improve the clinical outcome of these patients [5]. Therefore, there is a great need for the identification of biomarkers that could help reflect the severity and prognosis of CTD-ILD. Red blood cell distribution width (RDW) is a quantitative measure of anisocytosis, reflecting the variability of erythrocyte dimension. It is routinely measured by automated hematology analyzers and has been reported as a component of the complete blood cell count panels. Traditionally, RDW is primarily used in the investigation of the etiology of anemia [6]. In the past few years, RDW has been regarded as a useful prognostic indicator of numerous diseases, including critical illness [7], SARS-CoV-2 infection [8], and respiratory diseases such as nonsmall cell lung cancer (NSCLC) [9], chronic obstructive pulmonary disease [10], community-acquired pneumonia [11], and idiopathic pulmonary fibrosis (IPF) [12]. A previous study has confirmed that increased RDW is associated with CTD-ILD risk under various CTD backgrounds [13]. However, there are no studies on whether RDW could indicate the severity and prognosis of CTD-ILD. Therefore, in this study, we aimed to investigate the characteristics of RDW in CTD and CTD-ILD. In addition, we also explored the potential association of RDW levels at the time of diagnosis with the severity and survival of patients with CTD-ILD.

## 2. Materials and Methods

### 2.1. Study Population

This retrospective study included 310 patients who were diagnosed with systematic vasculitis (*n* = 18), SSc (*n* = 4), SLE (*n* = 30), SS (*n* = 103), RA (*n* = 16), DM (*n* = 45), PM (*n* = 3), MCTD (*n* = 41), and UCTD (*n* = 50), in Nanjing Drum Tower Hospital between February 2017 and February 2020. A total of 213 patients with ILD were included. 213 CTD-ILD patients were further divided into several radiological entities, including usual interstitial pneumonia (UIP) (*n* = 25), nonspecific interstitial pneumonia (NSIP) (*n* = 199), and organizing pneumonia (OP) (*n* = 86). It is worth mentioning that all patients with CTD-ILD or CTD without ILD did not use corticosteroids or immunosuppressant at baseline. We used American College of Rheumatology (ACR) criteria for the diagnosis of RA, SLE, and systematic vasculitis [14, 15]. The diagnosis of SSc was based on the LeRoy and Medsger criteria [16]. DM and PM were diagnosed using the Bohan–Peter criteria [17]. SS was diagnosed with the American-European criteria [18]. Patients were considered to have UCTD if they had evidence of polyarthritis not fulfilling ACR criteria or RP or nonspecific manifestations that did not meet ACR criteria for a specific rheumatic disease [19]. The diagnosis of MCTD was based on the Alarcon-Segovia criteria [20]. ILD was diagnosed on the basis of clinical presentation, physical examination, pulmonary function tests, and HRCT images. Patients who were diagnosed as CTD-ILD met the published guideline [21]. Exclusion criteria were as follows: (1) subjects had hematological disorders; (2) subjects overlapped with other diseases such as cerebrovascular disease, cardiovascular disease, hepatopathy, chronic obstructive pulmonary disease (COPD), pneumonia, pulmonary embolism, and pneumothorax; (3) subjects with an already known CTD or CTD-ILD.

### 2.2. Methods

Clinical information was obtained from the electronic medical record database of Nanjing Drum Tower Hospital. The clinical data included gender, age, smoking history (current smoking or previous smoking), pulmonary function tests, and laboratory findings at admission. Percent predicted forced vital capacity (FVC), percent predicted forced expiratory volume in one second (FEV1), and percent predicted diffusion capacity for carbon monoxide (DLCO) were included. Survival status was determined by reviewing the medical records or telephone follow-ups until September 2020.

### 2.3. Statistical Analysis

Continuous variables were tested for normal distribution by the Kolmogorov-Smirnov test. Continuous variables with a normal distribution were expressed as the mean ± standard deviation (SD), or they were presented as the median and interquartile range. Differences between the two groups were analyzed by *t*-test or the Mann–Whitney *U* test. Categorical variables were expressed as percentages and compared by the Chi-square test. Receiver operator characteristic (ROC) analyses were performed to calculate the area under the ROC curve (AUC) of RDW for the value of RDW for discriminating CTD-ILD from CTD without ILD and prognostic value of RDW in CTD-ILD patients. A cut-off value that ensured an optimum combination of sensitivity and specificity was calculated. The relationship between lung function parameters and RDW was also assessed by Pearson correlation analysis. Cox proportional hazard analysis was performed on potential prognostic factors. The Kaplan-Meier method was used to assess survival curves with GraphPad Prism version 7 (Graph Pad Software Inc., La Jolla, CA, USA). The log-rank test was used to evaluate the statistical significance of differences between the higher RDW and lower RDW groups. Data were analyzed using SPSS18.0 statistical software. *p* < 0.05 (two-sided) was considered to indicate statistical significance.

## 3. Results

### 3.1. Baseline Characteristics of Patients with CTD-ILD and CTD without ILD

Clinical characteristics and comparisons between 213 patients with CTD-ILD and 97 CTD patients are shown in Table 1. Male gender, smoking history (current smoking or previous smoking), and older age were more common in the CTD-ILD group (*p* = 0.001, *p* = 0.001, and *p* < 0.001, respectively). The levels of systolic pulmonary arterial pressure (sPAP) and hemoglobin were similar. Compared to the CTD group, patients with CTD-ILD had higher RDW and white blood cell (WBC) count (*p* < 0.001 and *p* = 0.001, respectively). There was no difference in the level of C-reactive protein (CRP), lactate dehydrogenase (LDH), serum total bilirubin (TBil), direct bilirubin (DBil), neuron-specific enolase (NSE), cytokeratin 21-1 (CYFRA21-1), and carcinoembryonic antigen (CEA).

### 3.2. ROC Curve Analysis for Discriminating CTD-ILD from CTD without ILD according to RDW

**Figure 1:**
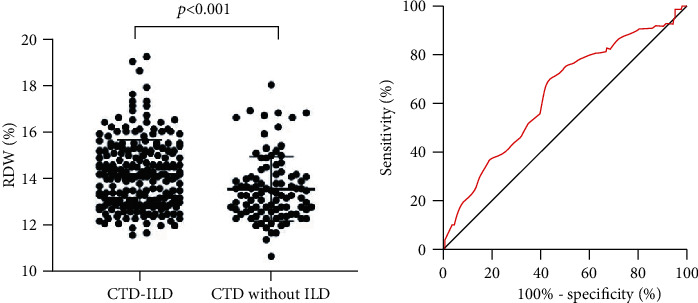
The value of RDW for discriminating CTD-ILD from CTD without ILD.

The RDW was compared between the CTD-ILD patients and controls. RDW was higher in the CTD-ILD patients than in CTD patients (14.20 ± 1.45 vs. 13.57 ± 1.38%, *p* < 0.001) (Figure 1(a)). The accuracy of RDW for discriminating CTD-ILD from CTD without ILD was then evaluated by receiver operating characteristic (ROC) analysis. The area under the ROC curve was 0.64 (95% CI: 0.57-0.70, *p* < 0.001) (Figure 1(b)). The cutoff value of RDW for discriminating CTD-ILD from CTD without ILD was 13.95% with their corresponding specificity (70.1%) and sensitivity (55.9%).

### 3.3. Correlations between RDW and Lung Function Parameters in CTD-ILD Patients

**Figure 2:**
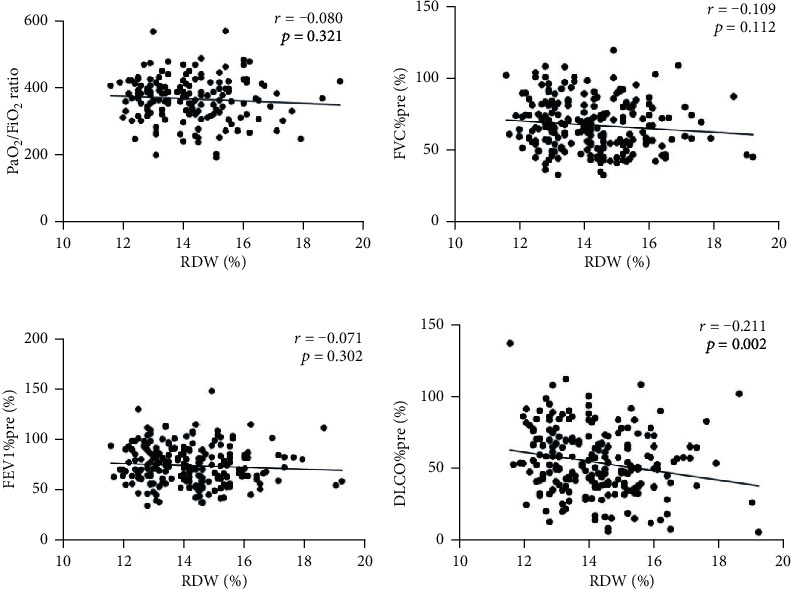
Correlations between RDW and lung function parameters in patients of CTD-ILD at baseline.

Correlation analyses showed that the increased RDW was significantly correlated with decreased DLCO%predicted (*r* = −0.211, *p* = 0.002) (Figure 2(d)). The relationships between RDW and PaO2/FiO2 ratio (oxygenation index), FVC%predicted, and FEV1%predicted in CTD-ILD patients at baseline were presented in Figures 2(a) – 2(c).

### 3.4. Association of RDW with Overall Survival of Patients with CTD-ILD

Cox proportional hazards models were used to examine the influence of RDW on the prognosis of patients with CTD-ILD. The univariate analysis showed that age, gender, sPAP, erythrocyte sedimentation rate (ESR), WBC count, RDW, LDH, carcinoembryonic antigen (CEA), and cytokeratin 21-1 (CYFRA21-1) were associated with survival in all 213 cases (all *p* < 0.05, respectively), while only gender and RDW were the independent risk factors for survival in these patients (HR 0.330, 95% CI 0.122-0.892, *p* = 0.029 and HR 1.495, 95% CI 1.210-1.846, *p* < 0.001, respectively) (Table 2).

**Figure 3:**
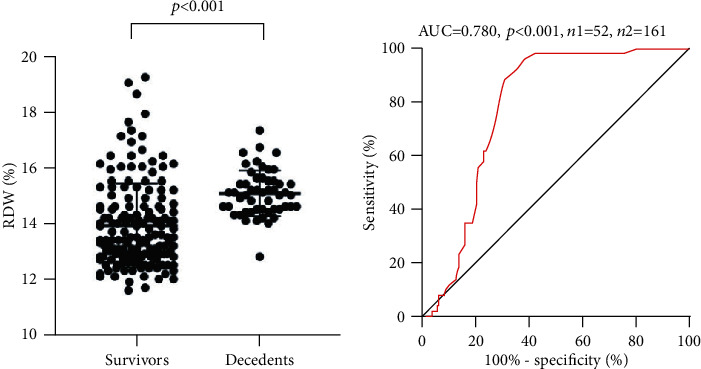
Prognostic value of RDW in CTD-ILD patients.

**Figure 4:**
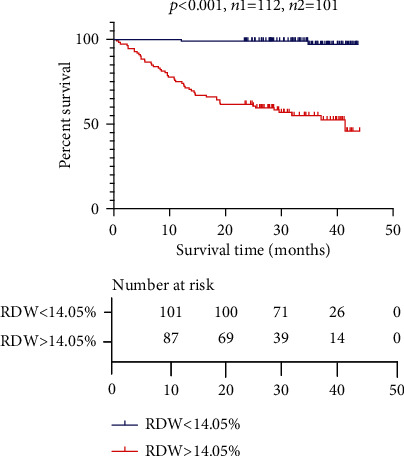
Survival of CTD-ILD patients in higher RDW group and lower RDW group.

To further investigate the value of RDW for predicting the prognosis of CTD-ILD, Figure 3(a) showed that RDW was significantly higher in decedents than survivors (*p* < 0.001). ROC analysis was conducted to determine the best cutoff value of RDW between the survivors and decedents (cutoff 14.05%, AUC 0.78 (95% CI: 0.72-0.84)) (Figure 3(b)). The CTD-ILD patients were divided into a higher RDW group (*n* = 112, RDW > 14.05%) and a lower RDW group (*n* = 101, RDW < 14.05%) to analyze the survival using the Kaplan-Meier method (Figure 4). The log-rank test showed a significant difference in survival between the two groups (*p* < 0.001). Up to September 2020, 50 CTD-ILD patients died in the group of patients with RDW > 14.05, and 2 CTD-ILD patients died in the group with RDW < 14.05, respectively. Among 50 decedents of CTD-ILD patients with higher RDW, 17 (34.0%) were DM, 16 (32.0%) were SS, 4 (8.0%) were systematic vasculitis, 4 (8.0%) were MCTD, 3 (6.0%) were RA, 3 (6.0%) were UCTD, 2 (4.0%) were PM, and 1 (2.0%) was SLE. The median survival time of decedents among the higher RDW group was 23.6 months. The mortality rate was higher in DM and PM (37.8% and 66.7%, respectively) among different CTD subgroups. Respiratory failure was the leading cause of death, where the incidence was 39.6% (19/48) in PM/DM-ILD and 15.5% (16/103) in SS-ILD. Respiratory failure was associated with acute exacerbation of ILD (AE-ILD) and pulmonary infection including cytomegalovirus and or EB virus, pneumocystis carinii pneumonia (PCP).

## 4. Discussion

In this retrospective study, we showed that patients with CTD-ILD had higher RDW compared with CTD without ILD patients. Moreover, there was a significant correlation between RDW and pulmonary function parameters indicating disease severity such as DLCO%predicted in CTD-ILD patients. Furthermore, our results also showed that CTD-ILD patients with relatively lower RDW had significantly longer overall survival than patients with relatively higher RDW. Thus, RDW was identified as possible significant prognostic predictor of CTD-ILD independent of any other risk factors.

CTD-ILD is a chronic lung disorder which is characterized by various patterns of inflammation and fibrosis. Clinically, patients with CTD-ILD can present with progressive dyspnoea, reduced gas exchange, and finally respiratory insufficiency of variable degrees. Based on the fact that CTD-ILD could have a significant adverse effect on quality of life and was a leading cause of mortality, there was a great importance of accurate diagnosis and appropriate clinical management [3]. Patients with SSc or PM/DM could have the relatively high prevalence and potential life-threatening course, highlighting the importance of early detection of ILD through regular chest HRCT. However, for other CTDs, such as RA, SS, and SLE, regular chest HRCT was not currently recommended due to the fact that these patients experienced stable or slowly progressive ILD [1]. Furthermore, respiratory failure often inhibited CTD-ILD patients from properly performing pulmonary function test (PFT). Considering the cost-effectiveness and readily access, RDW measurement by routine blood test would be a good alternative to evaluate the current status and severity of ILD. In our study, we found that patients with CTD-ILD had higher RDW than CTD without ILD patients. The cut-off value of RDW obtained from ROC curves could be helpful to distinguish CTD-ILD from CTD without ILD, which could be of great clinical importance.

Accurate assessment of the severity of CTD-ILD could help identify individuals more likely to benefit from and response to therapy. So far, it has been reported that several biomarkers such as Krebs von den Lungen-6 (KL-6), SP-D (surfactant protein-D), SP-A (surfactant protein-A), and serum B cell–activating factor (BAFF) may be useful for predicting the severity, therapeutic responsiveness, and prognosis of CTD-ILD [22, 23]. Moreover, there were researches studying tumor markers such as CEA and CYFRA21-1 that might reflect the severity and prognosis of ILD [24, 25]. In the present study, our results indicated that increased RDW was found in patients with CTD-ILD, in whom was inversely correlated with lung function including DLCO%predicted. Thus, RDW may reflect the severity of CTD-ILD patients. Nevertheless, using multivariate cox proportional hazard analysis, we identified RDW as an independent prognostic factor for CTD-ILD (HR = 1.495, *p* < 0.001). The best cutoff value of RDW to predict the survival of patients with CTD-ILD was 14.05%. Moreover, Kaplan-Meier survival curves of the patients with CTD-ILD indicated that the lower RDW group had a longer survival time than the higher group. Therefore, the baseline RDW was related to the prognosis of CTD-ILD; however, this hypothesis needs to increase the size of the sample for further confirmation. Compared with previous prognostic indicators, RDW offers a cheaper, simpler, and more convenient parameter for inclusion in routine blood examinations and follow-up.

RDW is a marker that quantifies the variation of individual red blood cell (RBC) volumes and can be measured quickly, cheaply, and easily through a routine CBC analysis. Several studies have investigated the correlation between elevated RDW and the morbidity and mortality of nonhematologic disorders, including heart disease, pulmonary disease, and cancer [26, 27]. In a study by Nathan et al., the RDW has been reported to be a readily available marker that may provide important prognostic information both at baseline and with serial change in patients with IPF [12]. Moreover, a recent study found that there was a significant correlation between elevated RDW at the time of hospital admission and increased mortality risk for patients with COVID-19 who received treatment [8]. The exact mechanism regarding the effect of RDW on adverse outcomes in various diseases remains unclear. Possible mechanisms may include the fact that elevated levels of RDW may reflect an underlying inflammatory state and oxidative stress [28]. Inflammatory cytokines affected bone marrow function and inhibited erythrocyte maturation which allowed juvenile erythrocytes to enter into the circulation, eventually leading to elevated RDW [29]. In addition, oxidative stress increased RDW by disrupting erythropoiesis and reducing red blood cell circulation half-life [30]. The pathogenesis of CTD-ILD has been reported to be involved with immune dysregolation, autoimmune processes, cell senescence, oxidative stress, and epithelial dysfunction [31]. Therefore, one potential mechanism that may account for the association of RDW with CTD-ILD is also inflammation and oxidative stress, which deserves further investigation.

Our study has several limitations. First, the cross-sectional study design limits the ability to infer causality between RDW and CTD-ILD. A longitudinal study in a larger population to validate the role of RDW in CTD-ILD would be required. Second, the ratio of CTD-ILD patients to CTD without ILD group was unbalanced towards ILD patients. Third, the pathogenesis of RDW in the occurrence of CTD-ILD remained unclear, and further basic research was needed to illustrate the specific mechanism. Finally, our sample size was limited and our results represent the experience of only a single centre. Therefore, it is uncertain whether these results are generalizable to other ethnic groups.

## 5. Conclusion

In summary, RDW may be clinically useful for distinguishing CTD-ILD from CTD without ILD. Our findings suggest that RDW may be used as a novel routine blood test-based biomarker to help reflect the severity and predict the survival of patients with CTD-ILD with the advantages of convenience, ease of accessibility, and low cost.

## Figures and Tables

**Table 1:** Baseline clinical features of all subjects.

Variable	CTD without ILD (*n* = 97)	CTD-ILD (*n* = 213)	*p* value
Gender (female, no. (%))	83 (85.6)	143 (67.1)	0.001
Smoking history (Y, %)	2 (2.1)	33 (15.5)	0.001
Age (years)	45.88 ± 17.76	58.84 ± 11.37	<0.001
sPAP (mmHg)	30.73 ± 7.38 (*n* = 44)	30.46 ± 7.05 (*n* = 171)	0.826
ESR (mm/h)	49.44 ± 33.18 (*n* = 84)	31.11 ± 24.62	<0.001
CPR (mg/L)	4.85 (2.80, 10.73) (*n* = 94)	4.50 (2.80, 8.80)	0.346
WBC count (10^9/l)	6.00 ± 2.98	7.11 ± 2.38	0.001
RDW (%)	13.57 ± 1.38	14.20 ± 1.45	<0.001
Hb (g/l)	138.75 ± 11.93	139.54 ± 13.85	0.813
MCV (fL)	91.50 ± 4.70	90.07 ± 4.04	0.020
LDH (U/L)	302.09 ± 200.87	276.24 ± 100.98	0.242
TBil (umol/l)	8.52 ± 7.91 (*n* = 93)	8.42 ± 3.15	0.907
DBil (umol/l)	1.80 (1.15, 2.80) (*n* = 93)	2.20 (1.70, 3.00)	0.800
CEA (ng/ml)	2.20 ± 1.21	2.24 ± 1.17	0.113
CYFRA21-1 (ng/ml)	4.58 ± 2.04	4.22 ± 2.19	0.131
NSE (ng/ml)	16.69 ± 6.74	16.51 ± 4.68	0.772

sPAP: systolic pulmonary arterial pressure; ESR: erythrocyte sedimentation rate; CRP: C-reactive protein; WBC: white blood cell; RDW: red blood cell distribution width; Hb: hemoglobin; MCV: mean corpuscular volume; LDH: lactate dehydrogenase; TBil: total bilirubin; DBil: direct bilirubin; NSE: neuron-specific enolase; CYFRA21-1: cytokeratin 21-1; CEA: carcinoembryonic antigen.

**Table 2:** Univariate and multivariate Cox proportional regression analysis of the relationships between clinical and biochemical parameters and prognosis of CTD-ILD patients.

Variables	Univariate cox model			Multivariate cox model		
	HR	95.0% CI	*p* value	HR	95.0% CI	*p* value
Age (years)	1.04	1.014-1.066	0.003	1.022	0.985-1.061	0.243
Gender (female)	0.569	0.329-0.984	0.044	0.330	0.122-0.892	0.029
Smoking history	0.564	0.296-1.075	0.082	2.130	0.599-7.576	0.243
sPAP (mmHg)	1.052	1.013-1.092	0.008	1.017	0.974-1.062	0.450
ESR (mm/h)	1.015	1.006-1.024	0.002	1.009	0.989-1.029	0.383
CPR (mg/L)	1.008	0.997-1.019	0.164	0.993	0.973-1.014	0.508
WBC count (10^9/l)	1.175	1.060-1.302	0.002	1.065	0.904-1.256	0.451
RDW (%)	4.251	3.145-5.745	<0.001	1.495	1.210-1.846	<0.001
LDH (U/L)	1.002	1.000-1.004	0.015	0.998	0.992-1.003	0.448
CEA (ng/ml)	1.065	1.023-1.109	0.002	1.029	0.969-1.092	0.349
CYFRA21-1(ng/ml)	1.135	1.032-1.249	0.009	1.044	0.879-1.239	0.625
NSE (ng/ml)	0.985	0.929-1.044	0.602	0.942	0.850-1.045	0.263
PaO2/FiO2 ratio (mmHg)	0.996	0.992-1.001	0.119	0.996	0.989-1.003	0.256

sPAP: systolic pulmonary arterial pressure; ESR: erythrocyte sedimentation rate; CRP: C-reactive protein; WBC: white blood cell; RDW: red blood cell distribution width; LDH: lactate dehydrogenase; NSE: neuron-specific enolase; CYFRA21-1: cytokeratin 21-1; CEA: carcinoembryonic antigen; PaO2/FiO2: oxygenation index.

## Data Availability

Data can be submitted by corresponding authors in case of a request.
